# Nanomaterial Exposure Induced Neutrophil Extracellular Traps: A New Target in Inflammation and Innate Immunity

**DOI:** 10.1155/2019/3560180

**Published:** 2019-02-28

**Authors:** Hang Yang, Tony N. Marion, Yi Liu, Lingshu Zhang, Xue Cao, Huifang Hu, Yi Zhao, Martin Herrmann

**Affiliations:** ^1^Department of Rheumatology and Immunology, West China Hospital, Sichuan University, Chengdu, Sichuan, China; ^2^Department of Microbiology, Immunology and Biochemistry, University of Tennessee Health Science Center, Memphis, USA; ^3^Department of Internal Medicine 3, Rheumatology and Immunology, Friedrich-Alexander University Erlangen-Nürnberg (FAU), Erlangen, Germany

## Abstract

Nanotechnology has become a novel subject with impact in many research and technology areas. Nanoparticles (NPs), as a key component in nanotechnology, are widely used in many areas such as optical, magnetic, electrical, and mechanical engineering. The biomedical and pharmaceutical industries have embraced NPs as a viable drug delivery modality. As such, the potential for NP-induced cytotoxicity has emerged as a major concern for NP drug delivery systems. Thus, it is important to understand how NPs affect the innate immune system. As the most abundant myeloid cell type in innate immune responses, neutrophils are critical for concerns about potentially toxic side effects of NPs. When activated by innate immune stimuli, neutrophils may initiate NETosis to release neutrophil extracellular traps (NETs). Herein, we have reviewed the relationship between NPs and the induction of NETosis and release of NETs.

## 1. Introduction

Nanotechnology has emerged as one of the most exciting industrial innovations worldwide [1, 2] in diverse areas of structural and material design and device and systems engineering. Nanoparticles (NPs) are defined as structures whose sizes are within the range from 1 to 100 nm in one, two, or three dimensions while nanomaterials are a group of small-scale substances which are applied to carry out their distinct properties in many kinds of fields, including but not limited to optical, magnetic, mechanical, and electrical engineering [3–5]. NPs also have the unique biological characteristic of high surface-to-volume ratio and small size. Due to their unique structural and size properties, they can easily penetrate molecular, cellular, and extracellular matrix barriers to reach most body systems. While NPs can be easily taken up by cells, they may also bind to cell surface proteins, initiate signaling, activate or inactivate the relevant cells, and in some cases cause unexpected cellular interactions [6, 7]. At present, environmental exposure and deliberate administration are two approaches by which NPs may be introduced. As the potential for NP exposure from inhalation, ingestion, and direct skin contact has increased [8, 9], nanotoxicology has emerged as a new type of toxicology to evaluate the safety of nanostructures and nanodevices [10].

The innate immune system is the first line of immune defense for mammalian and other eukaryotic hosts including mice and humans. Innate immunity includes both soluble proteins such as secreted cytokines and acute-phase and complement system proteins [11–14] and cells from the myeloid, lymphoid, and mast cell lineages [13–21]. The myeloid cells include granulocytes (neutrophils, basophils, and eosinophils), monocytes, macrophages, and dendritic cells [16–18]. Innate lymphoid cells (ILC) [19], natural killer cells (NK) [20], and to some extent *γδ* T cells [21] are the lymphoid representatives to innate immunity. Mast cells, although similar in many respects to granulocytes, are a distinct lineage of innate immune cells [15]. Cells from all of these cell lineages are the main effector cells in innate immune responses [22] to both pathogenic and nonpathogenic challenges through pattern recognition receptor (PRR) recognition of pathogen-associated molecular patterns (PAMPs) to initiate an inflammatory response [23]. Polymorphonuclear leukocytes and neutrophils (PMNs) are not only the most abundant leukocytes in the blood, up to 65% of white blood cells in humans, but also short-lived. PMNs are derived from a granulocyte-monocyte precursor in adult bone marrow [24] and account for more than fifty percent of hematopoietic activity. Each day, there are about 5 × 10^10^ PMNs released from bone marrow into the peripheral circulation [25, 26]. Due to the PMN's short lifespan, close to 24 hours, homeostatic control is essential to maintain relatively stable cell numbers in the circulation. Acute bacterial or fungal infection, for example, stimulates an immediate inflammatory response by the vascular endothelium and the migration of PMNs to the site of infection in response to local chemokines and local changes in endothelial integrins [27]. The recruited PMNs phagocytose and kill the potential pathogens. Upon phagocytosis of potential pathogens, PMNs initiate a respiratory burst to generate reactive oxygen species (ROS) that are bactericidal [28].

## 2. Critical Role of Nanoparticles in Immune Response and Inflammation

The effects of NPs on the immune system, especially the innate immune system, are critical to a thorough understanding of the physiological and pathophysiological consequences of NP exposure. Intentional or unintentional NP exposure will initiate engagement of cellular and soluble protein components of the innate immune system to activate intracellular and extracellular signaling cascades [9, 29, 30] in response to the NPs. Both extracellular and intracellular innate immune receptors, pattern recognition receptors (PRR), may be engaged and stimulated by NPs [31–33]. Likewise, proteins in serum, particularly those in the complement [34, 35] and kallikrein [36] systems, may be engaged by NPs. Whether the NP interaction gradually leads to stimulation or inhibition of innate immunity and or inflammation is determined by the physicochemical properties of the relevant NPs [37–41]. NPs such as sand, dust particles, or pollen are generally ignored by the immune system. On the other hand, when NPs engage PRR, the NPs may mimic pathogen-associated molecular patterns (PAMPs) and initiate an innate immune reaction [42–44].

Upon exposure to NPs, neutrophils may initiate an inflammatory response, secreting signaling chemokines and evoking downstream reactions [14]. The physical and chemical properties of NPs are major factors that may affect the innate immune response. Differences in size, size distribution, charge, surface area, reactivity, crystallinity, aggregation in relevant medium, composition, surface coating, method of synthesis, and impurities not only affect biodistribution and cellular uptake of NPs but also affect innate immune responses [9, 45–48]. It still remains controversial whether the toxicity of NPs originates from the NPs themselves, metal ions released by dissolution of the NPs, or a combination of both. Several studies demonstrate that the released metal ions are the major or even the only cause of their toxicity. Soluble NPs, such as ZnO and FeO that have higher surface ion dissolution, were reported to be more toxic than NPs with less surface ion dissolution, such as CeO_2_ and TiO_2_ [49–52]. Some studies have also indicated that size is an important determinant for toxicity and the inflammatory potential of NPs. Larger-size NPs with a smaller surface-to-volume ratio have higher dissolution of toxic ions and induce more inflammatory ROS production [53]. Shape and composition are also critical determinants for NPs' toxicity and inflammatory potential [54]. The NPs' surface composition influences NPs' interactions with cell membranes and surface receptors. More positively charged NPs have higher potential to induce inflammatory reactions [55]. Thus, if aggregated NPs are dissociated through sonication, the cytotoxicity and ROS production may increase on account of the increased solubility and ion dissolution [56]. However, several studies report that the major source of toxicity of NPs is derived from their particulate characteristics [57, 58]. Wang et al. [59] reported that ZnO NP toxicity was due solely to the released Zn ions, and CuO NP toxicity originated from both the released Cu ions and the CuO particles. Toxicities of Fe_2_O_3_, Co_3_O_4_, Cr_2_O_3_, and NiO were caused by the particulate characteristics of the NPs. In consideration of the above, medical use of NPs must consider how NPs' physical properties, especially solubility, affect toxicity.

Usually, neutrophils take up NPs through pinocytosis, macropinocytosis, clathrin/caveolar-mediated endocytosis, or phagocytosis. Both micropinocytosis and pinocytosis are nonspecific and related to immune response. When neutrophil PRR engage NP PAMPs, that engagement may initiate inflammasome-dependent neutrophil activation [31, 60]. Recently, NETosis, a new cell death specific to neutrophils, has become another significant way by which NPs may stimulate immune and inflammation response [61, 62]. Herein, we focus on the correlation and interaction between NPs and NETs in innate immune responses.

### 2.1. NP-Induced NET Formation in Inflammatory Response and Inflammation Resolution

Neutrophil extracellular traps (NETs), a network structure released during NETosis, consist of 15-17 nm chromatin strands decorated with as many as 20 different antimicrobial proteins and peptides including myeloperoxidase (MPO), neutrophil elastase (NE), proteinase 3 (PR3), cathepsin G, LL37, and histones 1, 2A, 2B, 3, and 4 [62]. Conventional suicidal NETosis is usually initiated by several stimuli (bacteria, viruses, and fungi) binding to neutrophil toll-like receptors (TLRs) [62, 63], which activate the endoplasmic reticulum to release stored calcium ions. Elevated calcium levels increase protein kinase C (PKC) activity, inducing NADPH oxidase to assemble into the functional phagocytic oxidase (PHOX) complex [64–66]. PHOX generates ROS that initiates nuclear and granular membrane rupture with subsequent chromatin decondensation and diffusion into the cytoplasm [64, 65]. The aforementioned neutrophil granular proteins and peptides attach to the cytoplasmic chromatin, and the complexes break through the plasma membrane and diffuse into the extracellular space as NETs [61, 67]. Vital NETosis is another pathway to release NETs induced by *Staphylococcus aureus* [68] and *Candida albicans* [69] via blebbing of the nuclear envelope and vesicular exportation. Consequently, this pathway preserves the integrity of the neutrophil plasma membrane [66, 68, 70]. Meanwhile, it still remains controversial whether and how suicidal NETosis and vital NETosis coexist. Recent data suggest that other immune cells such as mast cells [71], eosinophils [72], and macrophages [73] can also release extracellular traps. When NPs stimulate NETs, the NPs may be captured within the NETs in a phagocytosis-independent process [70]. Recent results indicate that NETs may function in the setting of noninfectious disease and its regulation [70]. Several kinds of NPs such as gold, silver, cationic lipid, polystyrene, nanodiamonds, and graphene oxide (GO) were found to trigger NETosis [37, 55, 74–78] (Table 1).

### 2.2. Gold Nanoparticles (AuNPs)

Gold nanoparticles (AuNPs) have great potential in diagnostics and therapeutic nanomedicine [79]. AuNPs are recognized as nonbiodegradable and mostly insoluble in biological media, and they cause activation of neutrophils by altering the surface charge density on neutrophil membranes [80, 81]. AuNPs function as excellent nanocarriers not only because of their small size, which is similar to cellular components, but also because of their biocompatibility. AuNPs larger than 10 nm in size have less cytotoxicity and are more biocompatible [82, 83]. Bartneck et al. [77] explored the interaction between gold NPs with diameters of 15–50 nm and neutrophils. They built a successful model library of AuNPs with different surface chemistries or different shapes and studied their effect on human primary peripheral PMNs. Accordingly, they use transmission electron microscopy (TEM) or electroless deposition to observe that neutrophils trapped AuNPs mostly within extracellular networks. NETosis was detected 15 minutes after AuNPs come in contact with neutrophils and progressively trapped more NPs with time. AuNPs in different shapes and modified surface properties such as cetyltrimethyl ammonium bromide (CTAB) and polyethylene oxide (PEO) were compared to determine how size and surface properties affect NET formation. From that research, they concluded that NP's surface chemical characteristic had only a slight effect on NET formation but had significantly more impact on the range and ratio of gold PMN aggregates. The positively charged CTAB- and PEO-NH_2_-coated AuNPs were more frequently located internally in the NETs than PEO-OH- or PEO-COOH-modified NPs. In this study, we also found that unless we use DNase-pretreated neutrophils before staining, gold PMN aggregates could be detected. Meanwhile, gold NPs remain in the structure. It proves that there are some proteins in the gold PMN aggregate structure that may not be influenced by DNase and still play a role in trapping NPs. Since DNA structure is the main component part and carries a net negative charge, positively charged particles of AuNPs trapped by NETs can be explained by electrostatic forces [77]. Ali et al. [84] did researches on gold nanorods (AuNRs). In this study, AuNRs showed ability to treat cancer [84]. Another study has investigated how AuNPs with a size of 60 nm induce the generation of free radicals that may be involved in NET formation [81].

### 2.3. Silver Nanoparticles (AgNPs)

Silver nanoparticles (AgNPs) are widely used in many fields such as electronics, biosensing, and food adjuvants. AgNPs may also be used in medical applications such as drug delivery because of their size and antimicrobial properties [85]; however, AgNPs do have significant dose-dependent cytotoxicity. Meanwhile, it still remains controversial whether AgNPs or silver ions (Ag^+^) have been attributed to the cytotoxicity, because the majority of cell culture studies are done in suspension that makes it difficult to differentiate between particle and soluble Ag^+^ effects [86, 87]. A study in which AgNP particle dissolution (and aggregation) in cell culture media was prevented by using an air-liquid exposure cell system did not cause cytotoxicity or induce the release of proinflammatory markers [88]. However, more experiments are needed to clarify the fate of intracellular AgNPs and Ag^+^. Several studies have evaluated the effects of AgNPs on neutrophils including NET formation in vivo and in vitro. Liz et al. [74] reported that 15 nm AgNPs (AgNP_15_) induce atypical cell death in neutrophils in a caspase-1- and caspase-4-dependent process. AgNPs also induced ROS and IL1*β* [89]. The atypical cell death was also inhibited by the antioxidant n-acetylcysteine indicating ROS dependency on the AgNP-induced atypical cell death. AgNPs also induced NETosis in adherent neutrophils that could not be inhibited by caspase-1 and caspase-4 inhibitors [74]. During AgNP-induced activation, the volume of neutrophils increased when the expression of the neutrophil surface marker CD16 remained the same unlike apoptotic neutrophils where the CD16 expression decreased [90, 91]. These changes were related to oxidative stress.

### 2.4. Cationic Lipid Nanoparticles

Solid lipid nanoparticles (SLNs), which are made up of solid crystalline lipids at room and body temperature, are among the colloidal nanosystems [92]. Nowadays, SLNs are commonly used in nanomedicine as drug carriers for a variety of medical treatments including cancer therapy, medical diagnosis, and tissue impairing [93]. Cationic SLNs (cSLNs) have been useful as carriers for DNA and RNA in promoting gene transfection and expression, respectively [94, 95]. Investigation into the possible roles for cSLNs in inflammation is still lacking. Hwang et al. designed a study examining the effect of cSLNs on human primary neutrophils and whether cSLNs can induce NETosis. As noted above, NETosis is initiated when the nuclear and granular membranes rupture with subsequent chromatin decondensation and diffusion into the cytoplasm [61, 67]. Their results indicated that oxidative stress, Ca^2+^ influx, and MAPK pathway signaling were essential to cSLN-induced NET formation. All these findings indicate the significance of cSLNs in the activation of neutrophils [75].

### 2.5. Carbon and Polystyrene Nanopowders

**Figure 1:**
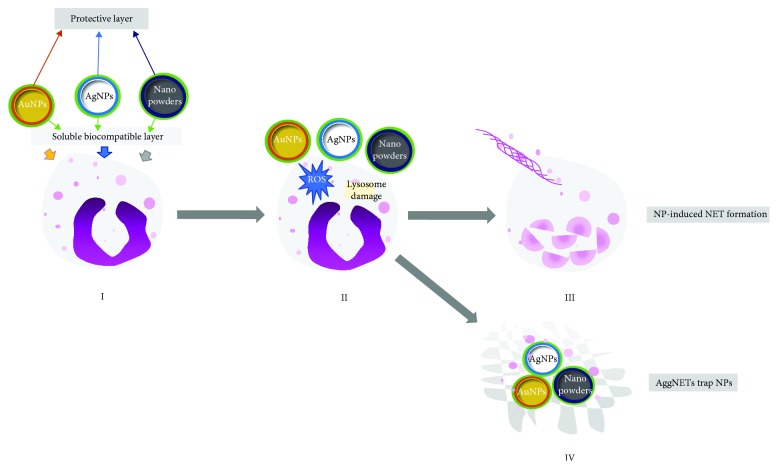
Several kinds of nanoparticles (gold nanoparticles, silver nanoparticles, nanopowders, etc.) can induce NET formation (I). Once the NPs contact the cellular membrane, cellular activation and/or cellular membrane damage can lead to lysosomal damage and ROS production (II). NP stimulation may induce histone deimination and chromatin decondensation resulting in the formation of NETs (III). Neutrophils may form aggNETs and trap NPs in order to eliminate and immobilize inflammatory particles (IV).

Muñoz et al. [76]. recently studied the interaction between neutrophils and carbon and polystyrene NPs. Environmental exposure to carbon NPs, including nanodiamonds, is unavoidable. Carbon NPs are a ubiquitous, necessary by-product of common procedures used in manufacturing and business including abrasive grinding and laser printing to carbon combustion that generates smoke. While NETs are induced by inflammation, aggregated NETs (aggNETs), which are generated under high neutrophil densities, may restrict and promote the absorption of inflammation [96]. Neutrophil NETs and aggNETs can capture and “neutralize” NPs in a size-dependent mechanism. When small NPs such as nanodiamonds (d) with a size of 10 nm (d10) and polystyrene beads (b) with a size of 40 nm (b40) were used to stimulate neutrophils, classical NET-like structures appear similar to those induced with PMA, whereas larger NPs (d_1000_ or b_1000_) did not induce NETs. Thus, there is a conclusion that both unipolar diamonds and polystyrene beads may induce NETs in a size-dependent way in vivo. This process activates a short-term inflammatory response and limits inflammation by immobilizing and entrapping NPs. They also got a conclusion that small-sized NPs may damage the cell molecular barrier and the function of cell membrane ion selectively. Oxidative stress and lysosomal damage are vital in NP-induced NETosis. The membranes were damaged by NPs and used for recycling in body systems firstly, then fused with primary lysosomes to form into phagolysosome. When lysosomes ruptured, the oxidative stress is being activated and the production of ROS is increasing beyond intracellular pathways. In order to prevent further tissue damage, neutrophils formed aggNETs to restrict and immobilize NPs that lead to an endpoint of inflammation [76] (Figure 1).

### 2.6. Oxidative Stress Is the Major Mechanism of NP-Induced NET Formation

A number of studies indicate that oxidative stress is a major pathway in NP-induced NET formation by nanoparticles such as AgNPs, cSLNs, and nanopowders [76, 77, 85, 95]. In classical PMA-induced NETosis, reactive oxygen species is a vital factor. Thus, there was a hypothesis that ROS is the major pathway in NP-induced NET formation. Research with AgNPs revealed that AgNP-induced NETosis could not be reversed by the inhibitors of caspase-1 and caspase-4 [74, 85]. IL-1*β*, an inflammatory cytokine, is also measured, and it was found that its expression is decreased due to the function of caspase-1 and caspase-4 inhibitors. ROS was assayed by flow cytometry and found to be increased by AgNPs. Therefore, it was concluded that AgNPs rapidly induced an atypical cell death in neutrophils by a mechanism involving caspase-1, caspase-4, and ROS [74]. In the research of cSLNs, Hwang et al. [97] found that cSLNs can activate neutrophils through respiratory and degranulation pathways. cSLNs induce a dose-dependent increase in superoxide anion production. Uptake of cSLNs activated Ca^2+^ channels and increased Ca^2+^ influx. Pretreatment with the Ca^2+^ influx inhibitor BAPTA-AM inhibited increases in Ca^2+^ influx and ROS induced by cSLNs [75]. Muñoz et al. concluded that both carbon and polystyrene nanopowders induced NETs by an oxidative stress-dependent mechanism. The NPs damaged neutrophil cell membranes and caused lysosome to rupture to activate the production of ROS and induce NETosis [76].

## 3. Conclusion and Perspective

Nowadays, nanoparticles have become widely used in engineering, vaccine carrying, and drug delivery due to their biochemistry and biocompatibility. The interaction between NPs and the innate immune system, especially neutrophils, is a vital area of research to be further pursued. Currently, neutrophils release NETs and trap sterile NPs and nonsterile pathogens as soon as they can, and NPs can be trapped due to their different biochemical properties. Further studies are needed to understand the interaction between NPs and NETs. Meanwhile, it is important to know the most vital properties of NPs in NETosis. Thus, NP-induced NET formation needs to be further investigated to figure out their physiological roles to utilize NPs well in nanomedicine.

## Figures and Tables

**Table 1:** Nanoparticles (NPs) that induce NETs.

NPs	Reference
Gold	[77]
Silver	[55, 74]
Cationic lipid	[75]
Polystyrene	[76]
Nanodiamonds	[76]
Graphene oxide	[78]
